# The modified lateral supraorbital approach for tumors of the petroclival junction extending into the anterior cerebellopontine area

**DOI:** 10.1007/s11060-016-2061-9

**Published:** 2016-02-17

**Authors:** Jaejoon Lim, Kyunggi Cho

**Affiliations:** Department of Neurosurgery, Bundang CHA Medical Center, CHA University College of Medicine, Yatap-dong 59, Seongnam, 463-712 Korea

**Keywords:** Petroclival meningioma, Trigeminal schwannoma, Combined petrosal approach, Modified lateral supraorbital approach, Cerebellopontine angle

## Abstract

Various surgical approaches for the removal of meningioma and trigeminal schwannoma in the petroclival junction (PCJ) and anterior cerebellopontine area (CPA) have been described previously. In this study, we compared the surgical outcomes of the combined petrosal approach and a modified lateral supraorbital (MLSO) approach and evaluated the reliability and safety of the MLSO approach. Fifty patients underwent surgical treatment using the combined petrosal or MLSO approach between 1996 and 2011. We retrospectively analyzed the clinical data and compared the two approaches. Among 50 patients, 27 patients underwent operation through the combined petrosal approach and 23 underwent operation through the MLSO approach. The operation time of the MLSO approach was significantly shorter than that of the combined petrosal approach (p = 0.03). There was no significant difference in the gross total resection rate between the two approaches (p = 0.67). After the operation, the improvement in Karnofsky performance score and Mean Glasgow outcomes scales were better in the MLSO approach, but without statistical significance (p = 0.723, p = 0.20 respectively). Complications occurred more often with the combined petrosal approach than with MLSO. Facial nerve palsy was the most common complication, followed by hearing difficulty. The frequency of these two complications was higher in the combined petrosal approach. Various tumors occurring in the PCJ and anterior CPA remain a challenging problem for neurosurgeons. The new modified approach of MLSO yielded good surgical results for these tumors compared to the combined petrosal approach. Therefore, the MLSO approach might be a good option for removal of tumors in the PCJ including anterior CPA.

**Electronic supplementary material:**

The online version of this article (doi:10.1007/s11060-016-2061-9) contains supplementary material, which is available to authorized users.

## Introduction

Central skull base lesions in the petroclival junction (PCJ) and anterior cerebellopontine area (CPA) can be challenging for surgeons to access because of their position and relation to the brainstem. Meningioma and trigeminal schwannoma are tumors that frequently occur in the petroclival area and the anterior CPA. These tumors are generated in narrow spaces and cause various symptoms by compressing the brainstem. It is very difficult to remove tumors in these areas because they are associated with important neurovascular structures including various cranial nerves as well as the brainstem. Several approaches are used to remove such tumors, including petrosal approach, retrosigmoid approach, fronto-orbito-zygomatic approach and other combined approaches [1–11]. In this study, we present a series of 50 consecutive patients with tumors of the PCJ or anterior CPA who were treated surgically with the combined petrosal approach or MLSO approach. We describe our experience, compare the outcome of each approach, and evaluate the reliability and safety of the MLSO approach.

## Materials and methods

### Patients

Fifty patients who underwent surgical treatment by combined petrosal approach or MLSO approach performed by one senior neurosurgeon and two well-trained neurosurgeons between 1996 and 2011 were included in this study. All patients had meningioma or trigeminal schwannoma in PCJ and anterior CPA.

### Surgical technique

#### Modified lateral supraorbital (MLSO) approach

**Fig. 1:**
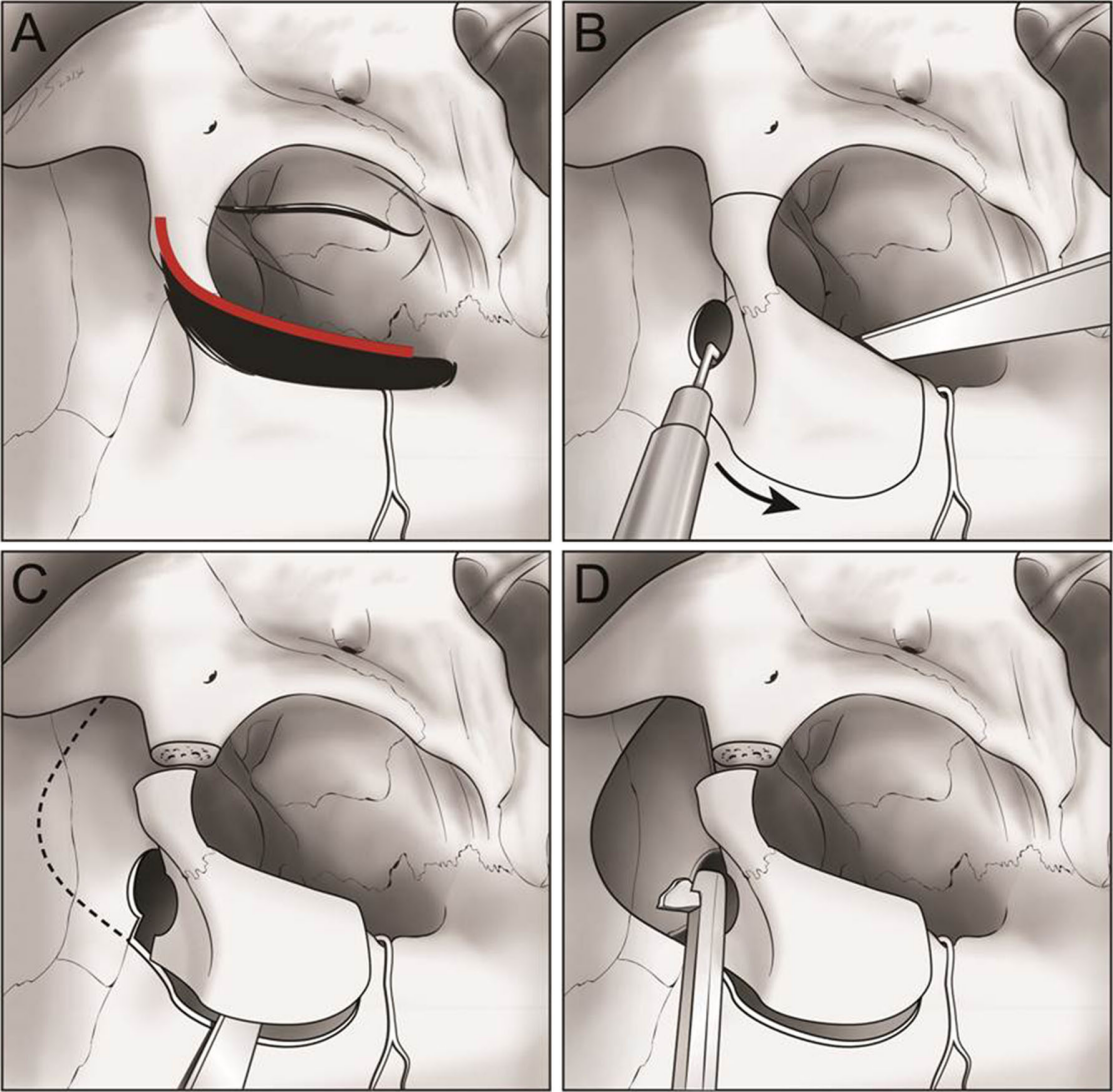
Modified lateral supraorbital (MLSO) approach. **a** Trans-eyebrow skin incision. **b** Craniotomy lines. **c** Free bone flap using a craniotome, including the supraorbital bone, frontozygomatic process, and frontal bone. **d** Temporal bone craniotomy using a rongeur and punch

**Fig. 2:**
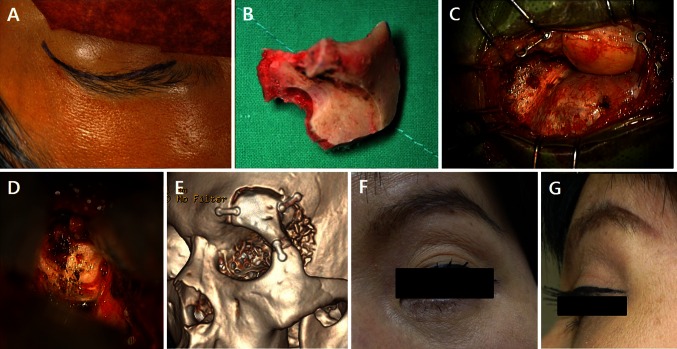
Surgical images. **a** Trans-eyebrow skin incision. **b** The bone flap. **c** The operation field. **d** Exposure of the petrous bone. **e** Postoperative bone-surface CT image. **f**, **g** Front and lateral view of the skin wound 3 months after surgery

The patient is positioned in a supine position with a Mizuho head holder and the head is elevated above the heart and turned to the contralateral side by 10–30°. The skin incision is located at the inferior edge of the eyebrow, starting from 0.5 cm medial to the mid-pupillary line and extending laterally to just behind the frontal process of the zygomatic bone and approximately 1 cm inferior laterally. A burr hole is drilled on the frontosphenoid suture and a craniotome is used to make a bone flap that includes the supraorbital bone, frontozygomatic process and frontal bone. The roof and lateral wall of the orbit are cut using an osteotome and the temporal bone is removed using a rongeur and punch. After exposure of the superior orbital fissure, the meningo-orbital band is transected to facilitate extradural access to the anterior clinoid process. The orbital roof is carefully removed and the optic canal is unroofed before performing anterior clinoidectomy. The outer layer of the cavernous sinus is peeled extradurally from anterior to posterior, exposing the inner membranous layer. The greater superficial petrosal nerve is the lateral landmark, the anteromedial margin of the eminencia arcuata is the posterior landmark, and the lateral margin of the porus trigeminus is the posterior landmark on the middle cranial fossa. After we confirm the anatomical landmarks of the Kawase triangle, the apex of the petrous bone is drilled out and then the petroclival junction and anterior CPA are opened. After removing the tumor, a cranial plate is used to fix the bone flap and a bone chip is used to fill the temporal craniectomy site (Figs. 1, 2).

### Data analysis

We retrospectively analyzed the clinical data, operation time, radiologic images, surgical outcomes and complications, and compared these data between combined petrosal and MLSO approaches. Patients received information about the surgical procedure and related complications with printed visual materials. Before the operation the researchers explained that the patients’ medical records would be used for research. Only the records of patients who provided consent were included in analysis.

Operation time was defined as the time from the initial skin incision to skin closure. Computerized tomography and magnetic resonance imaging were utilized for the initial diagnosis as well as assessment of resection rate. Gross total resection (GTR) was defined as the case in which no mass is visually evident and the tumor cannot be seen in postoperative images. Subtotal resection was defined as the case in which remnant mass volume is less than 20 % of initial tumor volume.

Preoperative and postoperative clinical conditions were assessed using the Karnofsky performance score (KPS). Functional outcome was evaluated using the Glasgow outcomes scale (GOS) with evaluation criteria as follows: I, death; II, vegetative state and severely disabled; III, moderately disabled; IV, mildly disabled; V, not disabled. Clinical and functional outcomes were investigated 1 year after the operation.

In addition, complications, morbidity and mortality related to the operation were examined, and postoperative tumor recurrence rates were compared.

## Statistical analysis

The independent *t* test was performed to compare the operation time between the combined petrosal approach and MLSO approach. The Whitney test was used to compare the operation time between the two approaches for each type of tumor. The Chi square test was carried out to compare improvement in KPS between approaches, and the independent *t* test was performed for comparison of GOS. SPSS ver. 18.0 was used for statistical analysis and a p-value <0.05 was considered significant.

## Results

### Patients

**Fig. 3:**
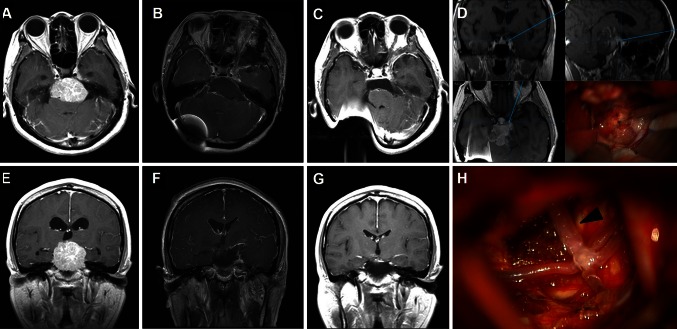
**a** and **e** Preoperative MR images of petroclival meningioma in a 45-year-old female patient who underwent ventriculoperitoneal shunt placement before tumor removal. **b** and **f** Immediate postoperative MR images show that the tumor was totally removed via the MLSO approach. **c** and **g** MR images show no recurrence of tumor 6 months after surgery. **d** The petroclival junction lesion and the tumor. **h** The basilar artery (*black arrow head*) was freely exposed after tumor resection

**Fig. 4:**
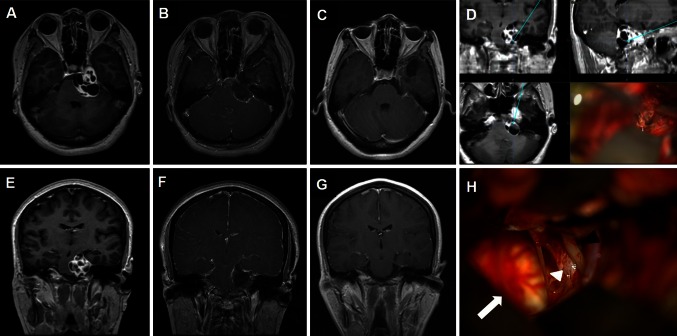
**a** and **e** Preoperative MR images of an anterior CPA schwannoma in a 46-year-old female patient. **b** and **f** Immediate postoperative MR images show that the tumor was totally removed via MLSO approach. **c** and **g** MR images show no recurrence of tumor 20 months after surgery. **d** The anterior CPA lesion and the tumor. **h** The pons (*black arrow*) was decompressed well with preservation of 3rd (*black arrow head*) and 4th (*white arrow head*) nerves after total resection of the tumor

A total of 50 patients with meningioma or trigeminal schwannoma in the PCJ and ACP area were included in the analysis. The mean age was 46.5 years, and 13 patients were male and 37 were female. The tumor volume of patients undergoing the combined petrosal approach and MLSO approach was 33.46 ± 17.0 cm^3^ and 32.79 ± 24.9 cm^3^, respectively, with no significant difference between the two groups (p = 0.925). More than 60 % of tumors were located in the anterior CPA for both approaches (Table 1). Major symptoms included headache, dizziness, facial hypoesthesia, and gait disturbance. Twenty-seven patients underwent operation by the combined petrosal approach, among whom 12 had meningioma and 15 had trigeminal schwannoma. Twenty-three patients underwent operation by the MLSO approach, nine with meningioma and 14 with trigeminal schwannoma (Table 1). Representative preoperative and postoperative MR images of meningioma and schwannoma are provided in Figs. 3 and 4.

**Table 1:** Characteristics of combined petrosal approach and MLSO approach

	Petrosal app.	MLSO app.
No. of patients	27	23
Male	5	8
Female	22	15
Mean age	43.30 ± 16.0	49.52 ± 18.3
Tumor type		
Meningioma	12 (44.4 %)	9 (39.1 %)
Schwannoma	15 (55.6 %)	14 (60.9 %)
Tumor volume (cm^3^)	33.46 ± 17.0	32.79 ± 24.9
Tumor location		
Anterior CPA	18 (66.7 %)	14 (60.9 %)
Petroclival junction	9 (33.3 %)	9 (39.1 %)
Clinical factors		
Hypertension	3 (11.1 %)	8 (34.8 %)
DM	2 (7.4 %)	4 (17.4 %)
Alcohol intake	6 (22.2 %)	8 (34.8 %)
Smoking	5 (18.5 %)	4 (17.4 %)

*Anterior CPA* anterior cerebellopontine angle, *DM* diabetes mellitus

### Surgical and clinical outcome

The mean operation time was 792.0 min for a combined petrosal approach and 454.1 min for an MLSO approach. Mean operation time of the MLSO approach was significantly shorter (p = 0.03). In patients with meningioma, the mean operation time was 780.0 min for the combined petrosal approach and a significantly shorter 570.6 min for the MLSO approach (p < 0.001). Similarly, the mean operation time of patients with trigeminal schwannoma was significantly longer for the combined petrosal approach compared to the MLSO approach (801.7 vs. 379.3 min, respectively; p < 0.001). GTR was carried out for 18 of 27 patients (66.7 %) who received combined petrosal approach and 14 of 23 patients (60.9 %) who underwent the MLSO approach (p = 0.67); subtotal resection was performed for the remaining patients. The GTR rate for the combined petrosal approach and the MLSO approach was 50.0 and 66.7 % respectively among patients with meningioma, and 80.0 and 78.6 % respectively for those with trigeminal schwannoma. KPS was improved after the operation in 74.1 % of patients who underwent the combined petrosal approach and 69.6 % of patients who underwent the MLSO approach; this difference was statistically insignificant (p = 0.723). Mean GOS measured 1 year after the operation was 4.4 for combined petrosal approach and 4.7 for MLSO approach.Although the MLSO approach showed a higher mean GOS, the difference was not statistically significant (p = 0.20) (Table 2).

**Table 2:** Surgical outcome and operation related complication according to surgical approach

	Combined petrosal app.	MLSO app.	p-value
Surgical outcomes			
Operation time (min)	792.0	454.1	0.03*
Meningioma	780.0	570.6	<0.001*
Schwannoma	801.7	379.3	<0.001*
GTR	18/27 (66.7 %)	14/23 (60.9 %)	0.67
Meningioma	6/12 (50.0 %)	6/9 (66.7 %)	0.66
Schwannoma	12/15 (80.0 %)	11/14 (78.6 %)	0.24
KPS change	20/27 (74.1 %)	16/23 (69.6 %)	0.723
GOS	4.4	4.7	0.20
Complications	10/27 (37.0 %)	6/23 (26.1 %)	
Facial palsy (House Brackmann)	5 (18.5 %)	2 (8.6 %)	

		Preop.	Postop.		Preop.	Postop.
	Case 1	I	II	Case A	II	III
	Case 2	II	IV	Case B	II	IV
	Case 3	II	III			
	Case 4	I	IV			
	Case 5	II	IV			
Hearing difficulty (MCL/SRT/WRS)	3 (11.1 %)			0 (0.0 %)		

		Preop.	Postop.	%
	Case I	40/10/100 % (40)	50/20/72 % (50)	
	Case II	55/25/88 % (55)	Hearing loss	
	Case III	30/14/86 % (30)	65/32/84 % (65)	
Visual field defect	0 (0.0 %)			1 (3.7)
6th nerve palsy	0 (0.0 %)			1 (3.7)
Hemiparesis	1 (3.7 %)			1 (3.7)
CSF leakage	1 (3.7 %)			0 (0.0)
Wound infection	0 (0.0 %)			1 (3.7)
Hydrocephalus	4 (14.8 %)			0 (0.0)

* Statistically significant

*MCL* (*dB*) most comfortable loudness level, *SRT* (*dB*) speech reception threshold, *WRS %* (*dB*) world recognition score

The mean follow-up duration was 82 months (range, 40–172 months) and there was no tumor recurrence. Tumor progression occurred in one patient with meningioma who underwent subtotal resection via the combined petrosal approach, but no surgical treatment or radiation treatment was performed because there were no new symptoms or neurologic deficits, and the change in tumor size was not large.

In our series, there were no operation-related mortalities. One patient who underwent surgery at the age of 96 died 13 months after the operation of old age while in a good recovery condition without any symptoms.

Operation-related complications occurred in a total of 16 patients (32 %), 10 patients (37.0 %) who underwent the combined petrosal approach and 6 (26.1 %) who underwent the MLSO approach. Among these complications, facial nerve palsy was most common, occurring in 7 patients (43.8 %), followed by hearing difficulty in 3 patients (18.7 %). The frequencies of these complications were higher in patients who underwent the combined petrosal approach (Table 2).

There were changes in cranial nerve function in 12 patients: hearing difficulty was observed in 3 patients; facial weakness in 7; 6th nerve palsy in 1; and aggravation of visual field defect compared to preoperative status in 1 patient. In 3 of 7 patients with facial weakness, the weakness was transient and subsequently recovered. A severe deficit of House Brackmann grade IV remained in the other 4 patients, and anastomosis was conducted for 3 of these patients. Hearing difficulty was not recovered in any of the patients. Sixth nerve palsy and visual field defect were transient and recovered 6 months after the operation. Two patients had hemiparesis, but they recovered within 1 month after the operation. One patient had CSF leakage and recovered after CSF drainage by lumbar puncture. One patient experienced wound infection and recovered after a 2-week course of antibiotics.

In addition, hydrocephalus occurred in four patients who had been operated on with the combined petrosal approach, all of whom received a shunt operation. In one patient who underwent a shunt operation for hydrocephalus before the tumor operation, shunt function was maintained after tumor operation.

## Discussion

To evaluate the usefulness of the MLSO approach for tumors in the PCJ extending into the anterior CPA area we compared the operation results, surgical complications and benefits of the petrosal and MLSO approaches through a retrospective study.

Complete removal of tumors is the primary goal of tumor surgery. However, tumors in the PCJ and anterior CPA area often extend into surrounding neurovascular structures and the brainstem, making complete removal difficult. Several surgical approaches for tumors in these regions have been developed over the past 40 years [1, 2, 4, 8, 10–14]. The petrosal approach is broadly divided into anterior, posterior and combined approaches [15]. The anterior petrosal approach reported by Bochenek in 1975 includes an extended middle fossa approach on internal auditory meatus and the cerebellopontine angle, and the anterior transpetrosal-transtentorial approach was first reported for the treatment of aneurysm of the lower basilar artery by Kawase and subsequently used for petroclival tumor resection [2, 6, 16, 17]. The posterior petrosal approach includes retrolabyrinthine, transcrusal, translabyrinthine, tranotic and transcochlear approaches [1, 5, 7, 18–21]. In the 1970s, King et al. described the translabyrinthine-transtentorial approach to the cerebellopontine angle, and Al-Mefty later described a retrolabyrinthine-transtentorial approach that could preserve hearing [1, 22]. Subsequently, several approaches have been reported to preserve hearing and facial nerve function. Sekhar described a procedure combining a presigmoid petrosal approach with partial labyrinthectomy and partial apicectomy [23]. Cho and Al-Mefty described a combined petrosal approach with preservation of hearing and facial functions, as well as wide petroclival exposure. This approach has the advantages of a wide view of the superior clivus, posterior cavernous sinus, and Meckel’s cave and provides minimal brain retraction and early access to feeding vessels. However, because the exposure is wider, the duration of the surgery and the risk of postoperative CSF leakage are increased [3].

A modified retrosigmoid approach was introduced by Samii for removal of tumors in the petroclival region [24]. Later, in addition to the basic advantages of the retrosigmoid approach, which minimizes drilling of petrous bone and handling of venous sinus, various modifications and newly developed methods were introduced to approach the middle fossa area with the aim of reducing hearing loss and minimizing cranial nerve damage [9, 25, 26]. In addition, if a tumor is extended above the tentorial notch, an orbitozygomatic approach should be additionally used to remove the tumor [12].

The selection of a surgical approach for tumors in the petroclival area depends on tumor extension, adjoining critical neural and vascular structures, and the relationship of the tumor with surrounding structures, including the petrous bone, clivus, tentorium, and cavernous sinus. The surgeon’s preference, experience, and the technique itself can also affect the decision of which surgical approach should be used. In this study, the combined petrosal approach was preferred when the tumor was located in the posterior, middle fossa or clivus region.

Anterior clinoidectomy and interdural dissection on the lateral wall of the cavernous sinus allows a wider area of exposure with some degree of medial mobilization of V3, which provides increased surgical access to CN VI at the Dorello canal, the upper two-thirds of the clivus, and the prepontine area (exposure of CNs II–VIII) [27]. Therefore, the MLSO approach was preferred for tumors located mainly in the posterior fossa extending through Meckel’s cave into the supra sellar, the cavernous sinus, CN II, CN III, and the 3rd ventricle.

Since the retrosigmoid approach requires excessive retraction of the cerebellum in PCJ extending into the anterior CPA area, this approach was excluded when conducting tumor removal in our series [14, 28].

We compared the surgical outcome between the combined petrosal approach and MLSO approach for tumors in the PCJ extending into the anterior CPA area to evaluate the reliability and safety of the MLSO approach. Operation time was significantly shorter for the MLSO approach compared to the combined petrosal approach, both in meningioma and trigeminal schwannoma patients. Previous studies reported an increase in occurrence of operation-related complications with an increase in operation time, and some studies suggested that the incidence of pulmonary complications was increased when the duration of anesthesia was long [29, 30]. Lamos-Luces et al. reported that the rate of surgical wound infection was related to operation time [30]. A study on surgical complications reported that the incidence of venous thromboembolism was increased according to operation time [31]. Therefore, short operation time can act as a positive factor in reducing the occurrence of operation-related complications.

In this study, GTR was carried out for 66.7 % of patients who received the combined petrosal approach and 60.9 % of patients who received the MLSO approach; this difference was statistically insignificant (p = 0.67). With consideration of the literature, the complete resection rate of petroclival meningioma ranges from 20 to 78 % [1, 10, 13, 14, 28, 32–35]. In meningioma patients of this study, the GTR rate was slightly higher with the MLSO approach, at 66.7 % compared to 50.0 % for the combined petrosal approach. This result was similar to or slightly higher than the results reported in the literature.

After the operation, KPS and GOS were measured for examination of clinical outcome and functional outcome. No significant difference was found between the MLSO approach and the combined petrosal approach in KPS and post-operative GOS measured 1 year after the operation.

Diverse complications related to surgery on tumors in the petroclival area have been reported. Among the larger series published over the past decade, the average reported mortality was 2 % (range 0–9 %), the rate of major morbidities was 23 % (range 7–39 %), permanent cranial nerve deficits occurred in 44 % of patients (range 29–76 %), and a poor functional outcome occurred in up to 17 % of patients [1, 10, 13, 14, 26, 28, 32–35]. In this study, there were 6 (26.1 %) cases of complications with the MLSO approach: facial palsy in 2 (8.6 %); visual field defect in 1 (3.7 %); 6^th^ nerve palsy in 1 (3.7 %); hemiparesis in 1 (3.7 %); and wound infection in 1 (3.7 %) patient. When compared with existing literature, this complication rate was relatively low [1, 10, 13, 14, 26, 28, 32–35]. The incidence of facial nerve palsy was higher in the patients who were treated with the combined petrosal approach (18.5 %) than in those treated with the MLSO approach (8.6 %). In particular, facial nerve palsy and hearing difficulty were most frequently observed among cranial nerve deficits, and both complications occurred more frequently with the petrosal approach. Among the 27 patients who were treated with the combined petrosal approach, 14 had serviceable hearing preoperatively and 3 of these had hearing deterioration postoperatively. Among the 23 patients who were treated with the MLSO approach, 16 patients had serviceable hearing preoperatively and none showed hearing deterioration postoperatively. Considering the anterior location of the tumor to the facial and cochlear nerves, the MLSO approach may prevent surgical damage of these nerves through its anterior approach. CSF leakage is an important complication that has been reported to occur in 2–17 % of cases [33, 36, 37]. In our series CSF leakage occurred in only 1 (2.0 %) patient, who underwent operation via the combined petrosal approach. Based on these results, mortality and morbidity rates in this study were similar to those reported in the literature. In addition to these clinical aspects, the important goals of surgical approaches are to minimize brain retraction, to obtain an appropriately sized bone flap for the operation, and to decrease the atrophy of muscles by reducing temporal muscle manipulation. In addition, a sufficient operation field should be secured. The biggest difference between these two approaches is that the MLSO approach involves concurrently generating the bone flap, including the orbital rim and performing temporal craniotomy from the zygoma suture line (Fig. 1). Removal of the orbital rim, temporal craniectomy and peeling of the outer layer of the cavernous sinus would allow an approach to the petroclival and anterior CPA areas from further inferior and anterior positions. Therefore, unlike the combined petrosal approach, which operates on petroclival tumors extended to the suprasellar or cavernous sinus in two stages, the MLSO approach can manipulate these tumors in a single stage.

In our series, there was no tumor recurrence and tumor progression occurred in only 1 patient with meningioma who underwent subtotal resection via the combined petrosal approach. The size of meningioma on follow-up imaging was increased, but there were no new symptoms or neurologic deficits, and the change in size was not large. Since there was no further change during 60 months of follow-up observation, reoperation or radiosurgery was not considered for this patient. Although there was only 1 case with progression during our study, it is necessary to investigate whether secondary surgery or radiation treatment is needed after following patients undergoing total resection or subtotal resection for as long as possible. According to Tao, the probability of recurrence is statistically high in cases with characteristics such as meningioma in the petroclival area, high histologic grade, low degree of tumor removal, irregular tumor shape and contrast medium enhancement [38]. It is therefore necessary to check for recurrence and progression through continuous follow-up. We periodically followed up our patients for a mean follow-up duration of 82 months (range 40–172 months).

Summarizing our findings, the MLSO approach has the following advantages for tumors in the PCJ and anterior CPA area.

1. Shortened operation time: It is not necessary to remove the labyrinthine bone in the MLSO approach. This reduced the operation time, and tumor removal was conducted without any difference in the degree of resection compared with other approaches. If there is no difference in the degree of tumor resection and morbidity is low, a short operation time helps reduce the recovery and hospitalization time of patients.
2. Preservation of hearing and facial nerve functions: Hearing difficulty and facial nerve malfunction, the major complications of a surgical approach to the PCJ and anterior CPA areas, can be prevented with the MLSO approach because of the anterior location of the tumor to the facial and cochlear nerves. There was no dysfunction in the cases of tumor removal by the MLSO approach. Also, the MLSO approach has the advantage of reducing lower cranial neuronal damage. Because this approach can access to the tumor in front of the nerves, it is easier to dissect and handle the tumor.
3. Low complications and low morbidity: CSF leakage is one important complication that may occur with existing approaches. After operation by the MLSO approach, the incidence of CSF leakage was 2–17 %, and there was no case with CSF leakage. It is less difficult to manage dura repair compared with other approaches.
4. Wide surgical exposure and good corridor: A surgical field similar to the surgical space of the orbito-zygomatic approach was obtained in addition to the existing familiar surgical space. Handling of the cavernous sinus through the intradural and extradural space is easy, and it can reach up to the upper 2/3 of the clivus area.
5. Smaller incision and no temporal muscle atrophy: Because of the remarkably small incision size and minimal temporal muscle dissection without any muscle cutting there was no post-operative temporal muscle atrophy, and patients showed very high satisfaction compared to other approaches.

The MLSO approach has the following limitations for tumors in the PCJ and anterior CPA area.

1. The free bone flap and craniectomy site is relatively small in the procedure for the MLSO approach. Therefore, if the tumor is accompanied by severe brain swelling the relatively small craniectomy site is not sufficient for decompression of brain swelling.
2. When the patient has frontal sinusitis there is a chance of infection during the craniotomy procedure. Therefore, the physician should check whether the patient is suffering from frontal sinusitis before the operation.
3. In cases where the tumor dura tail extends to the temporal area, which requires a large dura graft, it is difficult to suture with the MLSO approach.
4. In the MLSO approach it is hard to reach the upper two-thirds of the clivus area when the tumors are small because securing the route to the tumors is difficult due to surrounding structures.

This study has the following limitations:

1. It included a small number of patients.
2. It is a non-randomized and retrospective study.
3. It only targeted specific tumors of meningioma and trigeminal schwannoma.
4. All patients underwent surgery performed by a senior surgeon and two well-trained neurosurgeons as surgical assistants and the operation time might be affected according to the surgical assistant.

Accordingly, the MLSO approach needs to be further evaluated in a larger number of patients and for different types of tumors.

## Conclusion

Various tumors occurring in the PCJ and extending into the anterior CPA remain a challenging problem for neurosurgeons. The newly modified approach of MLSO achieved good surgical results compared to the combined petrosal approach for these tumors. Our data indicate that the MLSO approach might be a good option for removal of tumors of the PCJ extending into the anterior CPA, irrespective of whether they involve the cavernous sinus.

## Electronic supplementary material

Below is the link to the electronic supplementary material.

Video 1 A 45-year-old patient (Fig. 3) underwent operation for meningioma through MLSO approach

Video 2 A 46-year-old patient (Fig. 4) underwent operation for schwannoma through MLSO approach
